# Consumption of Dietary Fiber from Different Sources during Pregnancy Alters Sow Gut Microbiota and Improves Performance and Reduces Inflammation in Sows and Piglets

**DOI:** 10.1128/mSystems.00591-20

**Published:** 2021-01-26

**Authors:** Boshuai Liu, Xiaoyan Zhu, Yalei Cui, Wenjing Wang, Hua Liu, Zidan Li, Zhiguo Guo, Sen Ma, Defeng Li, Chengzhang Wang, Yinghua Shi

**Affiliations:** a College of Animal Science and Veterinary Medicine, Henan Agricultural University, Zhengzhou, China; b Henan Key Laboratory of Innovation and Utilization of Grassland Resources, Zhengzhou, China; Vanderbilt University Medical Center

**Keywords:** dietary fiber, inflammation, gut microbiota, sows, piglets, animal nutrition

## Abstract

In pregnant and lactating sows, metabolism and immunity undergo drastic changes, which can lead to constipation, abortion, and intrauterine growth restriction (IUGR) and reduce production performance. Dietary fiber can regulate animal gut microbiota, alleviate inflammatory responses, and improve performance. Here, 48 sows (Large × Landrace) were randomly allocated to groups including, control, and with alfalfa meal (AM), beet pulp, and soybean skin dietary supplementation for 60 days of gestation. The AM diet decreased IUGR, increased food intake during lactation, and promoted the reproductive performance and physical condition of sows. Further, the AM diet significantly reduced markers of intestinal permeability (reactive oxygen species and endotoxin) in sow serum, and of systemic inflammation (interleukin-6 [IL-6] and tumor necrosis factor alpha) in sow feces and serum, as well as piglet serum, while it increased the anti-inflammatory marker, IL-10, in sow serum and feces. The AM diet also significantly affected gut microbiota by increasing the relative abundance of proinflammatory bacteria, while decreasing anti-inflammatory bacteria. Moreover, the total short-chain fatty acid (SCFA) content was higher in feces from sows fed an AM diet, with butyric acid content significantly higher during lactation, than in controls. Sow performance was correlated with intestinal permeability, inflammation, and gut microbiota, which were also vertically transmitted to piglets. Our results are significant for guiding feed management in the pig breeding industry. Further, the “sows to piglets” model provides a reference for the effect of dietary fiber on the gastrointestinal function of human mothers and infants.

**IMPORTANCE** Although the direct effects of dietary fiber on gut microbiota composition have been studied extensively, systematic evaluation of different fiber sources on gut health and inflammatory responses of sows and their offspring has rarely been conducted. Excessive reactive oxygen species produced by overactive metabolic processes during late pregnancy and lactation of sows leads to increased endotoxin levels, disordered gut microbiota, decreased SCFA production, and secretion of proinflammatory factors, which in turn causes local inflammation of the gut, potential damage of the gut microbial barrier, increased gut permeability, increased blood endotoxin levels (resulting in systemic inflammation), and ultimately decreased sow and piglet performance. Our results showed that supplementation of the diet with alfalfa meal in mid and late pregnancy can reverse this process. Our findings lay a foundation for improving the gut health of sows and piglets and provide insights into the study of the gastrointestinal tract function in human mothers and infants.

## Abstract (precis)

Although the direct effects of dietary fiber on gut microbiota composition have been studied extensively, systematic evaluation of different fiber sources on gut health and inflammatory responses of sows and their offspring has rarely been conducted. Excessive reactive oxygen species produced by overactive metabolic processes during late pregnancy and lactation of sows leads to increased endotoxin levels, disordered gut microbiota, decreased SCFA production, and secretion of proinflammatory factors, which in turn causes local inflammation of the gut, potential damage of the gut microbial barrier, increased gut permeability, increased blood endotoxin levels (resulting in systemic inflammation), and ultimately decreased sow and piglet performance.

## INTRODUCTION

Sows and piglets are excellent animal models and have been widely used in biomedical research. Compared to rodents, sows and piglets are considered a superior model for studying the relationships in gut function of human mothers and infants (1). Pigs have many characteristics similar to humans, including digestive physiology, microbiota, and diet. These animals are suitable for a multitude of disease models, including diarrhea, gastrointestinal inflammatory disorders, necrotizing enterocolitis of neonates, and obesity, etc. (2). In large-scale pig production, sows and piglets are crucial to determining production levels and the economic benefits of pig farms, and the gestational, lactation, and newborn periods are core stages for feed management of sows and piglets in large-scale pig production (3). During pregnancy, sows undergo dramatic changes in physiological metabolism and immunity to ensure the implantation and development of embryos and pregnancy completion (4). In the mid and late periods of pregnancy, the levels of tumor necrosis factor alpha (TNF-α), interleukin-6 (IL-6), reactive oxygen species (ROS), and other proinflammatory factors increase significantly in the blood of sows (5) and are closely related to numerous diseases, including constipation, abortion, and intrauterine growth retardation (IUGR) (6, 7). Further, expression of the tight-junction protein, zonulin, is increased in sow guts, while bacterial lipopolysaccharide (LPS) entering the circulation through the gut barrier increased, and increased concentrations of bacterial endotoxins in the circulation can lead to metabolic endotoxemia, which is a potential mediator of inflammation (8–10). To initiate and maintain lactation, sows undergo complex metabolism and immune system changes that directly affect the development and growth of piglets (11). Therefore, reduction of inflammatory responses and ensuring normal metabolic and immune changes in sows during mid and late pregnancy and lactation is crucial for the performance of sows and their offspring (4). Gut microbiota has key roles in nutrient metabolism, immune development, protection against pathogens, and the pathogenesis of many chronic diseases in the host (12–14). Sow gut microbiota change dramatically during pregnancy and may be involved in metabolic processes in pregnant animals (15). Compared to the early stage of pregnancy, *Proteobacteria* and *Actinobacteria* are significantly increased in the sow gut during the late stage of pregnancy and have clear characteristics associated with increased risk of inflammation and energy loss (15). Short-chain fatty acids (SCFAs) are the main fermentation metabolites of gut microbiota and can stimulate cell signal transduction pathways via G protein-coupled receptor and upregulate the expression of Toll-like receptors (16). SCFAs also inhibit the activity of histone deacetylases (HDACs) and mRNA expression levels of the nuclear transcription factor, NF-κB, as well as downregulate the production of proinflammatory factors, to reduce gut inflammation (17). In addition, sow gut microbiota also participates in the immune development and maturation of their offspring. Transplanting microbiota colonized for a short period of time during the gestation period of a sow to germfree mice can promote the development of innate immunity in the gut and reduce inflammatory responses in their offspring via the activity of microbiota and metabolites (18). The SCFAs produced by sow gut microbiota can also be transferred to their offspring, where they promote the maturation and development of the immune system (19). Therefore, it is vital to understand the role of gut microbiota and their SCFA metabolites in the changes in inflammatory responses in sows during mid and late pregnancy and lactation.

Interest regarding the beneficial role of dietary fiber in regulating gut microbiota and physiological inflammatory responses is currently growing rapidly (20). Meanwhile, the advantages of dietary fiber in sows have gradually been exploited, e.g., by improving oocyte quality, increasing early embryo survival, improving lactation and weaning performance, and enhancing the vitality and uniformity of newborn piglets (21). This is primarily because dietary fiber can increase beneficial microbes in the gut, particularly lactobacilli, which aid in digestion and gut barrier function (22). In addition, SCFAs are the main fermentation products of dietary fiber and play an important role in gut health (23, 24). For example, acetate is an anti-inflammatory metabolite that maintains gut homeostasis, while butyrate helps to regulate gut permeability (25).

The effects of dietary fiber vary greatly due to their different sources and the variety and complexity of their chemical structures (26). Darroch et al. (27) added 20% soybean hulls and 0.3% psyllium to the diets of pregnant sows. Their results showed that soybean hull was more conducive to physical health maintenance in pregnant sows but had little effect on litter size. Cheng et al. (20) added combined soluble fiber from pregelatinized waxy corn starch and guar gum to the sows’ pregnancy diet, which significantly improved the developmental growth performance and gut function of 14-day-old suckling piglets. Zhuo et al. (28) found that insoluble fiber oat bran mixed with corn or soybean meal produced more SCFA by gut fermentation, which improved pig behaviors and reproductive performance. Although the direct effects of dietary fiber on gut microbiota composition have been studied extensively, systematic evaluation of different fiber sources (e.g., insoluble and soluble fiber sources) on gut health and inflammatory responses of sows and their offspring during mid and late gestation period has rarely been conducted. In this study, we evaluated the effects of adding different fiber sources, including soybean husk (SH), alfalfa meal (AM), and beet pulp (BP), to sow diets on growth performance, gut microbiota, gut permeability, and inflammation in sows and piglets. Our findings lay the foundation for screening specific fiber sources to improve the performance and gut health of sows and piglets and provide insights into the study of the gastrointestinal tract function in human mothers and infants.

## RESULTS

### Effects of supplementation in mid to late pregnancy with dietary fiber from different sources on the performance of sows and piglets.

Sows (*n* = 48; Large × Landrace) at 60 days of gestation were randomly allocated to groups as follows: control animals (CK) and animals with dietary supplementation using alfalfa meal (AM), beet pulp (BP), and soybean skin (SH). Assessment of sow backfat thickness during the reproductive cycle, including at gestation day 60 (G60d), G90d, lactation day 0 (L0d), and L21d, and sow feed intake by one-way analysis of variance (ANOVA) (Table 1) showed that there was no significant difference in average daily feed intake (*P* = 0.7620) and backfat (*P* = 0.6290 and *P* = 0.4240, respectively) among the treatment groups in mid to late pregnancy; however, in the lactation period, the average daily feed intake (*P* = 0.0320) of the AM group was significantly higher than that of CK, BP, and SH groups; in addition, the backfat loss of lactating sows showed a downward trend (*P* = 0.3750). Evaluation of sow reproductive performance demonstrated no significant differences in total litter size (*P* = 0.1380), live litter size (*P* = 0.1510), newborn body weight (*P* = 0.4430), or newborn litter weight (*P* = 0.1170) among the treatment groups; however, IUGR was significantly lower in the AM group than in the CK, BP, and SH groups (*P* = 0.0370) (Table 2). Further, we conducted a systematic study of piglet growth performance during lactation (L7d, L14d, and L21d) and found that individual body weight (*P* = 0.0700, *P* = 0.1580, and *P* = 0.0980, respectively) and litter weight (*P* = 0.0014, *P* = 0.1580, and *P* = 0.0980, respectively) of piglets in the AM, BP, and SH groups showed an increasing trend compared to the CK (Table 3). These data indicate that AM intake during mid to late pregnancy can increase feed intake and reduce IUGR in lactating sows.

**TABLE 1:** Effects of different fiber sources on fat thickness and feed intake of sows

Description	Mean ± SD^*a*^			
	CK	AM	BP	SH
Day 60 in gestation (mm)	19.82 ± 1.00	19.83 ± 1.35	19.83 ± 1.16	19.68 ± 0.84
Day 90 in gestation (mm)	19.54 ± 0.78	19.77 ± 1.22	19.83 ± 1.08	19.73 ± 0.61
Before parturition (mm)	20.84 ± 2.37	20.43 ± 1.43	20.72 ± 1.35	19.77 ± 0.80
After weaning (mm)	19.38 ± 1.54	20.53 ± 0.94	19.67 ± 2.11	19.15 ± 2.23
Gain during gestation (mm)	1.12 ± 1.76	0.65 ± 0.74	0.95 ± 2.83	0.22 ± 1.01
Loss during lactation (mm)	1.43 ± 3.78	–0.09 ± 0.16	1.20 ± 1.87	0.62 ± 2.44
Pregnancy feed intake (kg)	2.44 ± 0.27	2.37 ± 0.26	2.35 ± 0.39	2.46 ± 0.38
Lactation feed intake (kg)	7.75 ± 0.66^B^	8.76 ± 0.44^A^	7.80 ± 0.64^B^	7.66 ± 0.59^B^

a Data for the control (CK), alfalfa meal (AM), beet pulp (BP), and soybean skin (SH) groups are presented. The data were evaluated by one-way ANOVA, and significant differences between means were assessed by using Duncan’s test. Differences in the superscript letters for peer data indicate that a difference is significant (*P* < 0.05). The lack of a superscript letter means that all differences were nonsignificant (*P* > 0.05).

**TABLE 2:** Effects of different fiber sources on reproductive performance of sows

Description	Mean ± SD^*a*^			
	CK	AM	BP	SH
Total born piglets	13.11 ± 2.85	12.13 ± 3.31	10.44 ± 4.28	13.11 ± 2.62
Live-born piglets	12.67 ± 2.83	12.00 ± 3.42	10.11 ± 4.28	12.56 ± 2.46
IUGR (%)	9.48 ± 0.07^A^	2.08 ± 0.04^B^	11.21 ± 0.08^A^	8.94 ± 0.06^A^
Body wt of newborn piglets (kg)	1.35 ± 0.17	1.37 ± 0.12	1.40 ± 0.17	1.42 ± 0.17
Litter wt at birth (kg)	16.81 ± 2.42	16.49 ± 4.86	14.04 ± 5.73	17.72 ± 3.79

a Data for the control (CK), alfalfa meal (AM), beet pulp (BP), and soybean skin (SH) groups are presented. The data were evaluated by one-way ANOVA, and significant differences between means were assessed by using Duncan’s test. Differences in superscript letters for peer data indicate that a difference is significant (*P* < 0.05). The lack of a superscript letter means that all differences were nonsignificant (*P* > 0.05).

**TABLE 3:** Effects of different fiber sources on piglet performance

Description	Mean ± SD^*a*^ (*n* = 11)			
	CK	AM	BP	SH
Litter wt (kg)				
Day 0	19.48 ± 2.24	19.42 ± 2.02	19.44 ± 2.89	19.53 ± 1.23
Day 7	39.82 ± 1.74	39.87 ± 4.33	43.53 ± 4.61	42.76 ± 4.16
Day 14	54.90 ± 3.77	54.91 ± 2.38	57.59 ± 5.25	57.51 ± 6.39
Day 21	76.16 ± 5.80	82.56 ± 1.15	79.64 ± 6.66	75.93 ± 7.60
Avg daily gain	2.83 ± 0.29	3.16 ± 0.09	3.01 ± 0.20	2.82 ± 0.34
				
Body wt (kg)				
Day 0	1.38 ± 0.20	1.38 ± 0.18	1.38 ± 0.26	1.39 ± 0.11
Day 7	3.62 ± 0.16	3.62 ± 0.39	3.96 ± 0.42	3.89 ± 0.38
Day 14	4.99 ± 0.34	4.99 ± 0.22	5.24 ± 0.48	5.00 ± 0.66
Day 21	6.92 ± 0.53	7.51 ± 0.10	7.24 ± 0.61	6.90 ± 0.69
Avg daily gain	0.26 ± 0.03	0.29 ± 0.01	0.27 ± 0.01	0.26 ± 0.03

a Data for the control (CK), alfalfa meal (AM), beet pulp (BP), and soybean skin (SH) groups are presented. Eleven animals were included in each group. The data were evaluated by one-way ANOVA, and significant differences between means were assessed by using Duncan’s test. Differences in superscript letters for the peer data indicate that a difference is significant (*P* < 0.05). The lack of a superscript letter means that all differences were nonsignificant (*P* > 0.05).

### Effects of AM diet on gut permeability and inflammatory responses in sows and piglets.

**FIG 1:**
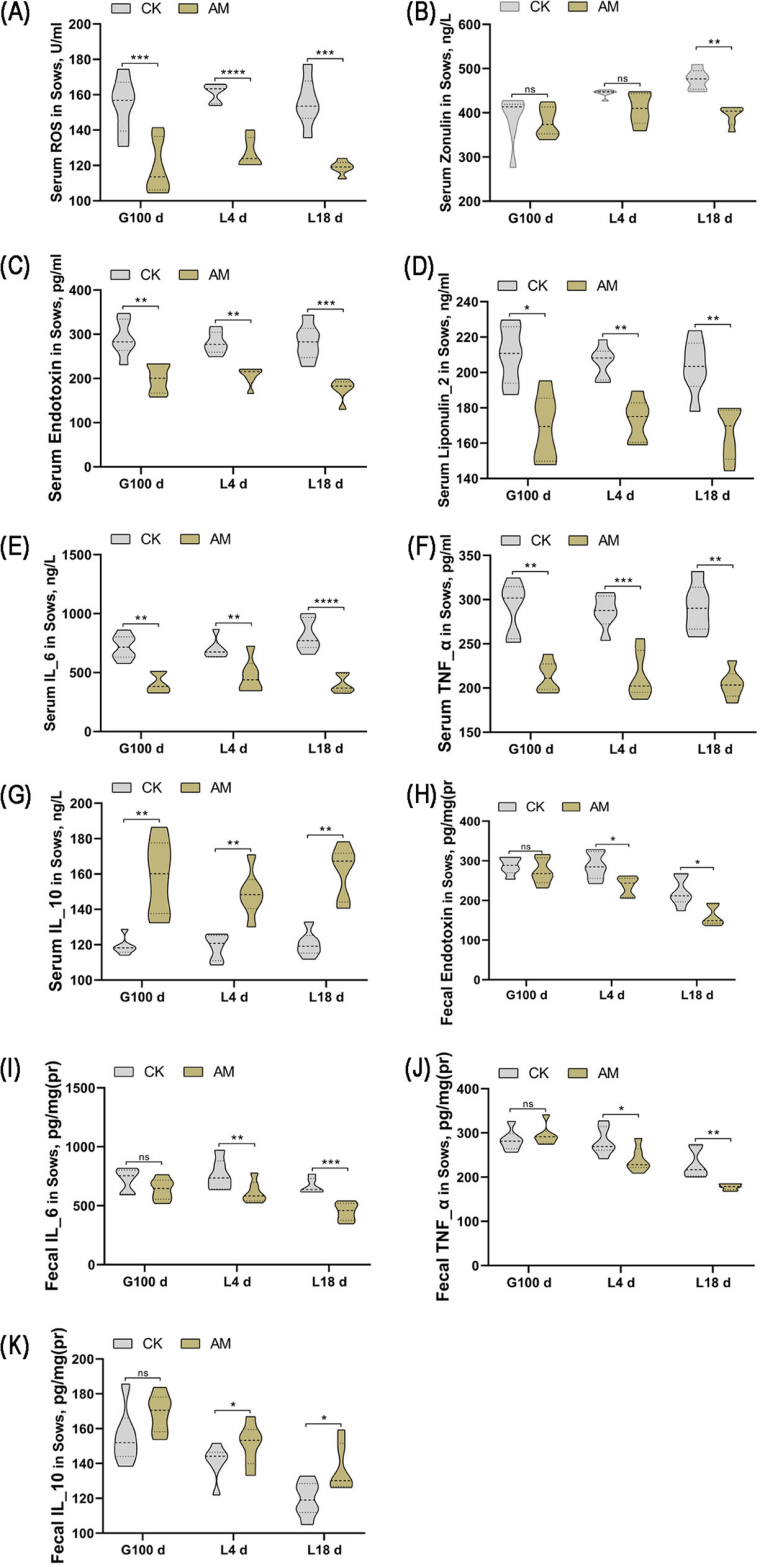
Effect of alfalfa meal diet on serum ROS (A), serum zonulin (B), serum endotoxin (C), serum lipocalin-2 (D), serum IL-6 (E), serum TNF-α (F), serum IL-10 (G), fecal endotoxin (H), fecal IL-6 (I), fecal TNF-α (J), and fecal IL-10 (K) levels of sows. CK, control group; AM, alfalfa meal group. The data were evaluated by one-way ANOVA, and significant differences between means were assessed by using Duncan’s test. *, 0.01 < *P* ≤ 0.05; **, 0.001 < *P* ≤ 0.01; ***, *P* ≤ 0.001; ns > 0.05.

Given our findings that AM supplementation could reduce IUGR, we next conducted a systematic evaluation of three biomarkers related to gut permeability (ROS, endotoxin, and zonulin) by one-way ANOVA at G100d, L4d, and L18d in sows with dietary supplementation with AM compared to controls. Compared to CK sows, the ROS (*P* = 0.0004, *P* < 0.0001, and *P* = 0.0010, respectively) and endotoxin (*P* = 0.0100, *P* = 0.0018, and *P* = 0.0007, respectively) levels were significantly lower at G100d, L4d, and L18d in serum samples from sows fed an AM diet (Fig. 1A and C). Further, serum levels of zonulin at L18d were significantly lower than those in the CK (*P* = 0.0014) (Fig. 1B). Together, these data suggest that AM intake in mid to late pregnancy reduces sow gut permeability. Next, we examined four biomarkers associated with gut inflammation in sows, IL-6, lipocalin-2, TNF-α, and IL-10. In terms of systemic inflammatory responses, we found that serum levels of IL-6 (*P* = 0.0014, *P* = 0.0010, and *P* < 0.0001, respectively), lipocalin-2 (*P* = 0.0122, *P* = 0.0039, and *P* = 0.0044, respectively), and TNF-α (*P* = 0.0012, *P* = 0.0008, and *P* = 0.0084, respectively) were significantly reduced, while those of IL-10 (*P* = 0.0040, *P* = 0.0025, and *P* = 0.0014, respectively) were significantly increased in sows fed a AM diet at G100d, L4d, and L18d, compared to controls in the CK (Fig. 1D to G). In sow feces, endotoxin levels were significantly lower at L4d and L18d (Fig. 1H). Evaluation of gut inflammatory responses demonstrated that levels of IL-6 (*P* = 0.0010 and *P* = 0.0017, respectively) and TNF-α (*P* = 0.0052 and *P* = 0.0084, respectively) were significantly reduced at L4d and L18d in fecal samples from sows fed with an AM, while those of IL-10 (*P* = 0.0130 and *P* = 0.0443, respectively) were significantly increased (Fig. 1I, J, and K).

**FIG 2:**
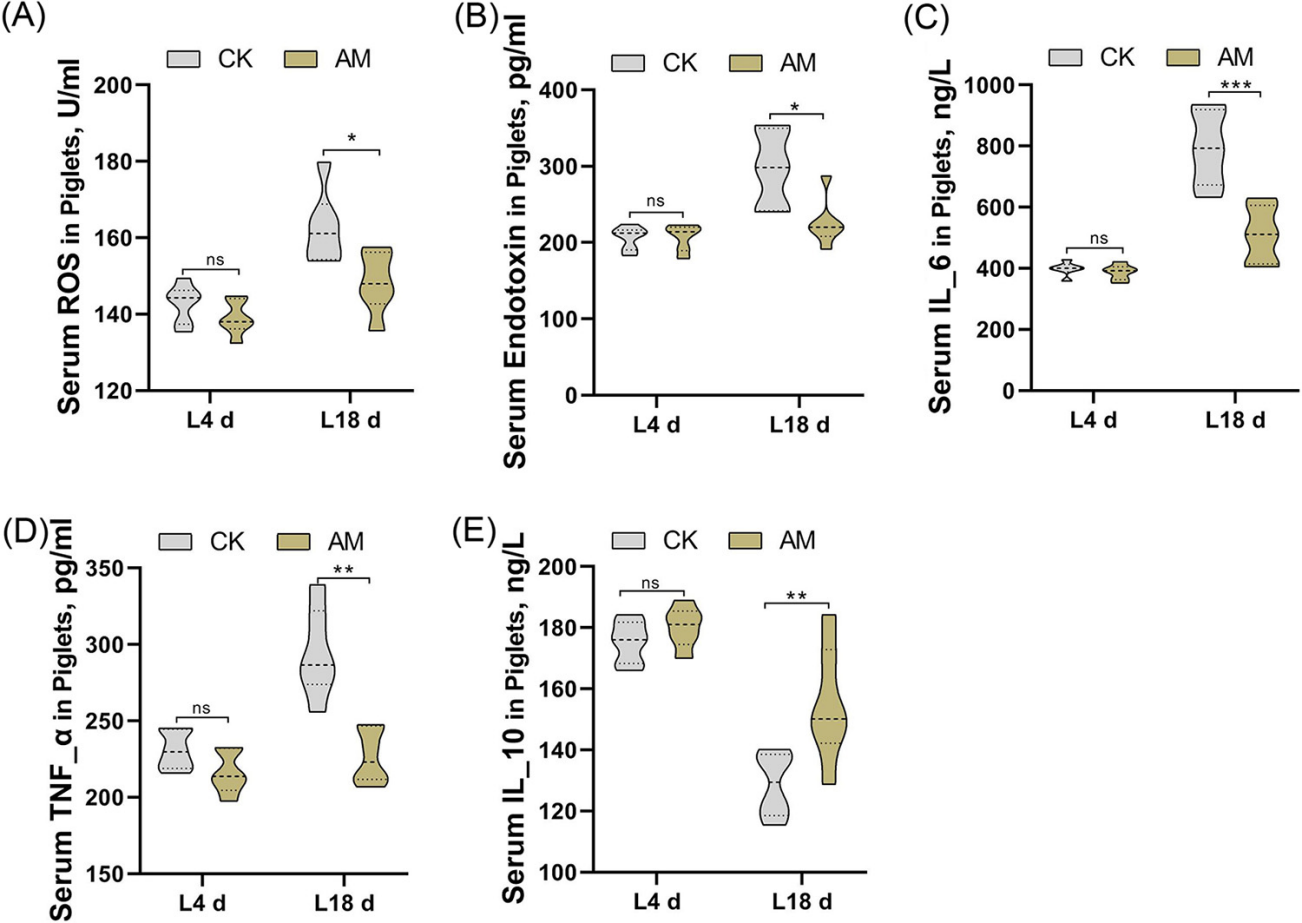
Effect of alfalfa meal diet on serum ROS (A), serum endotoxin (B), serum IL-6 (C), serum TNF-α (D) and serum IL-10 (E) levels of piglets. CK, control group; AM, alfalfa meal group. The data were evaluated by one-way ANOVA, and significant differences between means were assessed by using Duncan’s test. *, 0.01 < *P* ≤ 0.05; **, 0.001 < *P* ≤ 0.01; ***, *P* ≤ 0.001; ns, *P* > 0.05.

Further, we assessed these biomarkers by one-way ANOVA in the sera of piglets to understand whether feeding sows with an AM supplemented diet is related to gut permeability and systemic inflammatory responses in suckling piglets. We found no significant differences at L4d. At L18d, the serum levels of ROS (*P* = 0.0207), endotoxin (*P* = 0.0211), IL-6 (*P* = 0.0001), and TNF-α (*P* = 0.0067) were significantly reduced in AM group, while the serum levels of IL-10 (*P* = 0.0094) were significantly increased (Fig. 2). These results indicated that sows with alfalfa meal supplementation had lower levels of gut permeability biomarkers and lower levels of inflammatory markers in sows and piglets.

### An AM diet regulates changes in sow gut microbiota composition and microbial metabolites.

**FIG 3:**
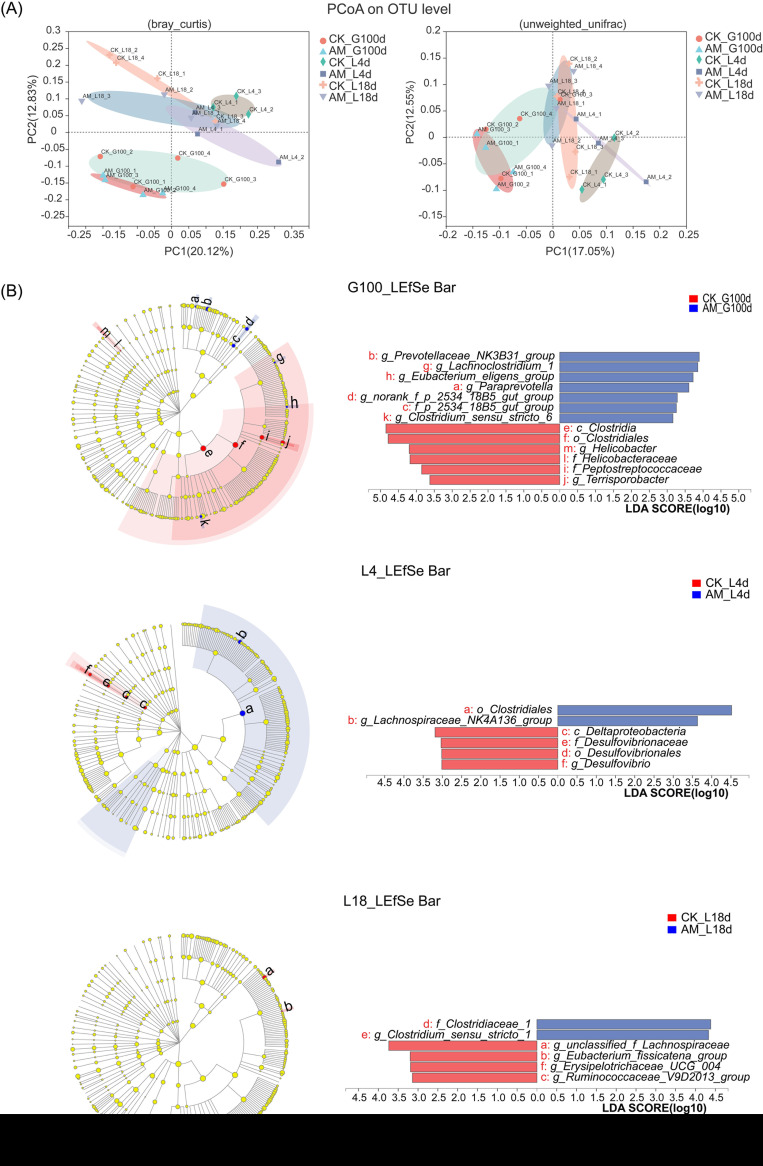
AM diet regulates the changes of gut microbiota composition in sows. (A) Principal-component analysis of OTU level by Bray-Curtis and unweighted-UniFrac distance; (B) LEfSe analysis determined by one-against-all (less strict). CK, control group; AM, alfalfa meal group.

The microbiota in sow fecal samples were analyzed at three time points (G100d, L4d, and L18d) by deep sequencing of the bacterial 16S rRNA gene V3-V4 region (see Fig. S1A in the supplemental material). The AM diet had no effect of on the Shannon (*P* = 0.1626, *P* = 0.5106, and *P* = 0.2304, respectively) and Chao (*P* = 0.2505, *P* = 0.8903, and *P* = 0.5451, respectively) indices by a Wilcoxon rank sum test in sow fecal microbiota at any time point (G100d, L4d, or L18d) (see Fig. S1B and C). Community composition at the phylum level indicated that indicated that the dominant microbiota at the three stages were *Firmicutes* (61.7 to 73.7%), *Bacteroidetes* (18.3 to 27.7%), *Spirochaetae* (3.6 to 7.6%), and *Proteobacteria* (0.5 to 4.5%) (see Fig. S1D), while dominant genera were *Clostridium_sensu_stricto_1* (5.7 to 13.2%), *norank_f_Bacteroidales_S24-7_group* (5.5 to 9.1%), *Terrisporobacter* (3.8 to 9.9%), *Christensenellaceae_R-7_group* (3.2 to 8.7%), and *Lactobacillus* (1.5 to 13.7%), among others (see Fig. S1E). Principal-component analysis by Bray-Curtis and unweighted-UniFrac distance showed that there were significant differences in microbiota at G100d, L4d, and L18d between the CK and AM groups (Fig. 3A). Further linear discriminant analysis effect size (LEfSe) analysis by “one-against-all” (less strict) to evaluate differences between the two groups showed that, at G100d, there was a significant increase in the relative abundance of *Prevotellaceae_NK3B31_group*, *Lachnoclostridium_1*, *Eubacterium_eligens_group*, *Paraprevotella*, *norank_fs_p_2534_18b5_gut_group*, and *Clostridium_sensu_stricto_6* and reduction in the relative abundance of *Helicobacter*, *Terrisporobacter* in animals fed the AM supplemented diet compared to the CK. At L4d, there was a significant increase in the relative abundance of *Lachnospiraceae_NK4A136_group* and a reduced relative abundance of *Desulfovibrio* in the AM diet group compared to the CK. Finally, at L18d, the AM diet led to a significant increase in the relative abundance of *Clostridium_sensu_stricto_1* and a reduction in *unclassified_f_Lachnospiraceae*, *Eubacterium_fissicatena_group*, *Erysipelotrichaceae_UCG_004*, and *Ruminococcaceae_V9D2013_group* relative to the CK (Fig. 3B). These results indicate that feed supplemented with AM during pregnancy significantly changes the gut microbiota composition in sows.

10.1128/mSystems.00591-20.1 FIG S1 AM diet regulates changes in gut microbiota composition in sows. (A) Sobs index of OTU level; (B) Shannon index of OTU level; (C) Chao index of OTU level; (D) microbiota community at the phylum level; (E) microbiota community at the genus level. CK, control group; AM, alfalfa meal group. *, 0.01 < *P* ≤ 0.05; **, 0.001 < *P* ≤ 0.01; ***, *P* ≤ 0.001. Download FIG S1, JPG file, 0.9 MB. Copyright © 2021 Liu et al. 2021 Liu et al. This content is distributed under the terms of the Creative Commons Attribution 4.0 International license.

**FIG 4:**
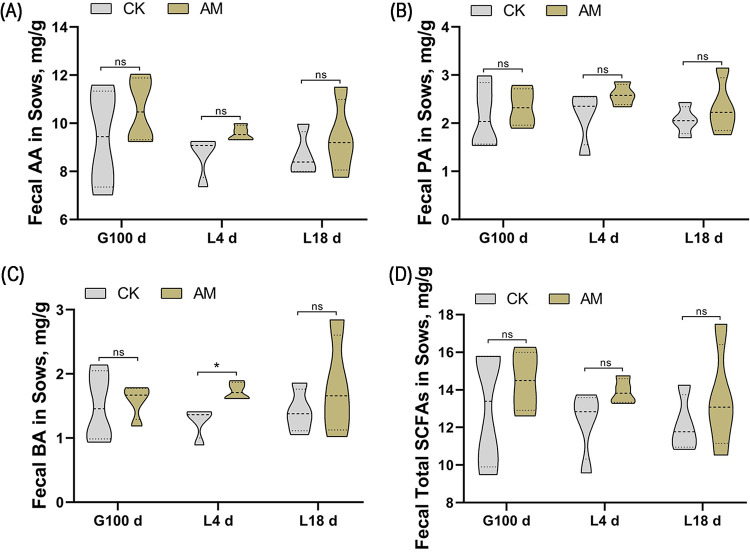
Effect of alfalfa meal diet on SCFAs fermentation of sows. (A) Fecal amino acids (AA); (B) fecal propionic acid (PA); (C) fecal butyric acid (BA); (D) fecal total SCFAs. The data were evaluated by one-way ANOVA, and the significant differences between means were assessed by using Duncan’s test. *, 0.01 < *P* ≤ 0.05; **, 0.001 < *P* ≤ 0.01; ***, *P* ≤ 0.001; ns, > 0.05.

To analyze the effect of an AM diet on gut microbial metabolism in sows during pregnancy, we next studied SCFAs by one-way ANOVA in sow excrement at three time points: G100d, L4d, and L18d (Fig. 4). The results showed that compared to the CK, all SFCAs in the feces of sows fed an AM supplemented diet had an increasing trend. There was no statistically significant except butyrate of L4d (*P* = 0.0360).

### Sow performance is related to gut permeability and inflammatory responses, which influence the health and growth of piglets.

Spearman correlation analysis by Euclidean distance found that sow performance was correlated with gut permeability and inflammation (Table 4), and IUGR was positively correlated with serum lipocalin-2, IL-6, and TNF-α and significantly negatively correlated with serum IL-10.

**TABLE 4:** Correlation between IUGR, lactation feed intake, and inflammatory factors of sows

Description	Correlation^*a*^							
	Serum lipocalin-2	Serum IL-6	Serum TNF-α	Serum IL-10	Fecal IL-6	Fecal TNF-α	Fecal IL-10	Serum ROS
IUGR (L4d)	0.864*	0.914*	0.926**	–0.923**	0.773	0.363	–0.616	0.840
Lactation feed intake (L18d)	–0.649*	–0.638*	–0.695*	0.685*	–0.745**	–0.398	0.201	–0.806**

a The data were evaluated by Spearman correlation analysis of the Euclidean distance. *, Mean significant correlation (*P* < 0.05); **, mean extremely significant correlation (*P* < 0.01).

Spearman correlation analysis by Euclidean distance found that sow feed intake during lactation was negatively correlated with serum lipocalin-2, IL-6, and TNF-α, as well as fecal IL-6 and serum ROS, and significantly positively correlated with serum IL-10. Gut permeability was correlated with inflammation in both sows and piglets (Table 5). In piglets, serum IL-10 was positively correlated with sow serum IL-10 and significantly negatively correlated with sow fecal TNF-α. Further, piglet serum TNF-α levels were positively correlated with serum IL-6, TNF-α, and ROS, as well as fecal IL-6 in sows, and significantly negatively correlated with piglet weaning body weight. Serum IL-6 in piglets was positively correlated with serum lipocalin-2, IL-6, TNF-α, and ROS in sows, while there was no significant correlation between serum ROS in piglets and sow inflammatory factors or piglet weaning body weight.

**TABLE 5:** Correlation between inflammatory factors of piglets and sows and weaning weight (L18d)

Piglet group	Correlation (sow group)^*a*^								
	Serum lipocalin-2	Serum IL-6	Serum TNF-α	Serum IL-10	Fecal IL-6	Fecal TNF-α	Fecal IL-10	Serum ROS	21d wt
Serum IL-10	–0.547	–0.698	–0.589	0.836*	–0.809	–0.885*	0.799	–0.630	0.324
Serum TNF-α	0.705	0.887*	0.877*	–0.691	0.822*	0.750	–0.693	0.832*	–0.866*
Serum IL-6	0.951**	0.910*	0.900*	−0.627	0.69	0.712	−0.735	0.825*	−0.730
Serum ROS	0.537	0.719	0.661	−0.467	0.635	0.517	−0.605	0.746	−0.662

a The data were evaluated by Spearman correlation analysis of the Euclidean distance. *, Mean significant correlation (*P* < 0.05); **, mean extremely significant correlation (*P* < 0.01).

### The composition of gut microbiota of sows regulated by an AM supplemented diet is related to their gut health.

**FIG 5:**
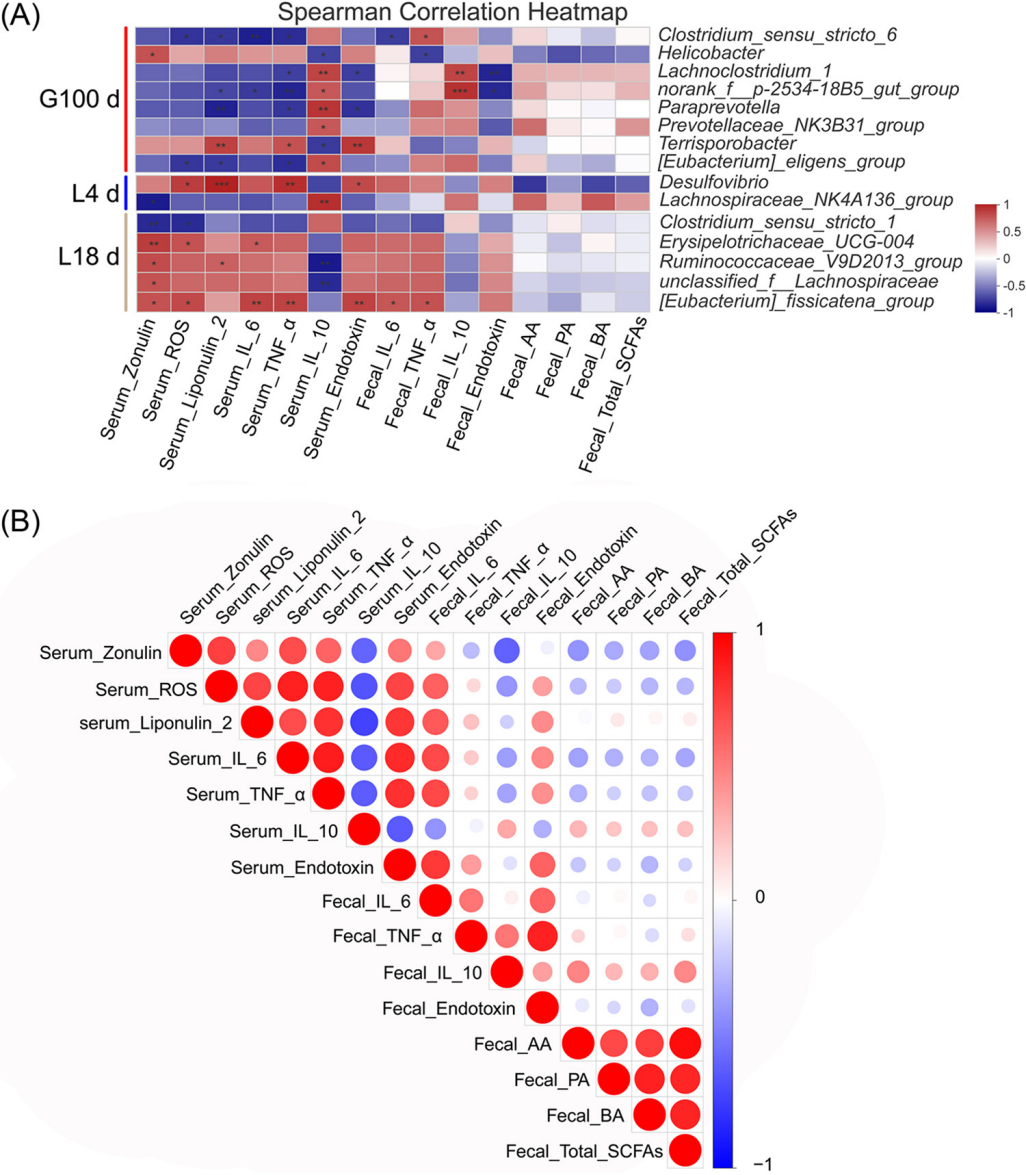
Association and model predictive analysis. (A) Correlation between gut microbiota and host markers by Spearman correlation analysis. (B) Correlation among host markers by Spearman correlation analysis. *, 0.01 < *P* ≤ 0.05; **, 0.001 < *P* ≤ 0.01; ***, *P* ≤ 0.001. Red indicates a positive correlation; blue indicates a negative correlation.

As shown in Fig. 5A, analysis of the correlation between microbiota differing according to LEfSe and metabolic indices in the gut tract of sows at each stage showed that the *Prevotellaceae_NK3B31_group* was significantly positively correlated with serum IL-10, while the *norank_f_2534-18b5_gut_group* was significantly positively correlated with serum IL-10 and fecal IL-10 and negatively correlated with serum lipocalin-2, IL-6, and TNF-α. *Terrisporobacter* was positively correlated with serum lipocalin-2, TNF-α, and endotoxin and negatively correlated with serum IL- 10. The *Lachnospiraceae_NK4A136_group* was positively correlated with serum IL-10, and *Clostridium_sensu_stricto_1* was significantly negatively correlated with serum zonulin and ROS levels. The *Ruminococcaceae_V9D2013_group* was positively correlated with serum zonulin and ROS and negatively correlated with serum IL-10, and the *Eubacterium_fissicatena_group* was positively correlated with serum zonulin, ROS, TNF-α, and endotoxin, and fecal IL-6. The *Norank_f_2534-18b5_gut_group* was significantly positively correlated with fecal IL-6 and negatively correlated with fecal IL-10. There were significant positive correlations between *unclassified_f_lachnospiraceae* and serum zonulin and ROS. In addition, the concentrations of acetic acid and butyric acid were positively correlated with the anti-inflammatory bacteria of the *Lachnospiraceae_NK4A136_group* and negatively correlated with the inflammatory bacterium, *Terrisporobacter*.

In addition, a series of correlation analyses between gut permeability, gut or systemic inflammatory responses, and metabolite markers (Fig. 5B) in sows revealed that serum ROS concentration was positively correlated with serum IL-6, TNF-α, endotoxin, and fecal endotoxin and negatively correlated with serum IL-10. The serum TNF-α concentration was positively correlated with serum IL-6, and serum endotoxin concentration was positively correlated with serum IL-6 and TNF-α levels. The concentration of fecal endotoxin was positively correlated with serum zonulin, endotoxin, IL-6, and TNF-α. The concentration of acetic acid in feces was positively correlated with that of propionic acid, while butyric acid concentration in feces was negatively correlated with ROS and IL-6 in serum and positively correlated with acetic acid and propionic acid in feces. The concentration of total SCFAs in feces was positively correlated with those of acetic acid, propionic acid, and butyric acid.

## DISCUSSION

The immune health status of reproductive sows directly affects overall pig productivity (3). During pregnancy, sow metabolism is enhanced, which manifests as an increase in appetite, digestive capacity, weight gain, and storage of numerous nutrients, to meet the requirements of fetal development. In the later stages of pregnancy, in addition to dietary energy, sows cease fat deposition and mobilize stored fat and energy during pregnancy, which is transferred to the mammary gland for milk synthesis. The metabolism and immune changes in sows during pregnancy and lactation affect the development and growth of fetuses, and disruption of these adaptive changes may lead to premature birth or even abortion (4). According to statistical analyses, in commercial genetic lines, the prebirth loss of piglets is around 30 to 50% (29). Therefore, it is crucial to reduce inflammatory responses in sows during mid and late pregnancy and lactation and to ensure that the normal metabolic immune changes occur in the sow reproductive cycle.

Some studies have shown that dietary fiber supplementation not only promotes satiety in sows but also improves sow feed intake during lactation (28, 30, 31); however, the effects of dietary fiber supplementation from different sources are inconsistent and may be closely related to the physicochemical properties and fermentability of dietary fiber (20, 27, 28, 32, 33). Here, we found that the addition of different fiber sources to the diet of sows in mid and late gestation affected the performance of both sows and piglets, with AM in particular significantly reducing the IUGR rate, increasing feed intake during lactation, and improving sow and piglet performance. AM is rich in insoluble fiber but also contains a small amount of soluble fiber, which can be fermented in the foregut segment, while the insoluble fiber can be slowly fermented in the hindgut, and has beneficial effects throughout the intestine (34). Dietary fiber can prevent and treat gut inflammation induced by a high-carbohydrate and low-fiber western diet in mice by restoring the damaged gut mucous layer (35). In the case of long-term or indirect dietary fiber deficiency, the gut microbiota resort to the use of mucosal glycoprotein secreted by the host as a nutrition source, leading to the erosion of the mucosal barrier. This results in increased pathogenic bacterial invasion of the mucosa and aggravation of physiological inflammatory responses (36). Prefeeding mice with inulin can reduce the gut inflammatory response and Smad7 expression after infection with Citrobacter rodentium and promote host protective immune responses by affecting the NF-κB and Smad7 signal transduction pathways (37); however, longitudinal studies on the dynamic changes in inflammation in sows during mid and late pregnancy and lactation are insufficient. In a recent study, sows that were in mid and late gestation and lactating were found to have symptoms of metabolic syndrome, mainly characterized by low-level inflammation and metabolic disorder (38). We assessed the gut permeability, gut and systemic inflammatory responses, and metabolic changes in sows fed AM. During the middle and late gestation and lactation periods in sows fed with AM, we found that three biomarkers of gut permeability (ROS, endotoxin, and connexin) and markers of inflammation (IL-6, lipocalin-2, and TNF-α) were decreased, which indicated that AM in the sow’s diet reduced gut permeability and decreased gut and systemic inflammation. Further, Spearman correlation analysis showed that there was a significant positive correlation between IL-10 in piglets and sows and that TNF-α expression in piglets was positively correlated with IL-6 and TNF-α expression in sows and negatively correlated with piglet weaning body weight. Moreover, sow feed intake during lactation was negatively correlated with lipocalin-2, IL-6, TNF-α, and ROS and significantly positively correlated with IL-10. These findings suggest that the improvements in performance observed in sows fed with an AM diet and their piglets are related to the alleviation of gut or systemic inflammatory responses and the improvement of physical health. Intrauterine developmental retardation remains a major problem in pig production because the associated low birth weight leads to high preweaning morbidity and mortality and permanent growth and developmental retardation. Improving the nutritional status of sows in the middle and late stages of pregnancy can effectively enhance the uniformity of embryos, thereby reducing changes in embryo development at the placenta stage and intraluminal fetal weight variation in the later stages of pregnancy (21, 39). Therefore, providing a balanced diet for sows, ensuring normal metabolic immune changes during the reproductive cycle, and reducing physiological inflammatory responses, particularly in the middle and late stages of pregnancy, are important for ensuring proper placental nutrient transport and, ultimately, improving piglet uniformity. In the present study, Spearman correlation analysis showed that IUGR was positively correlated with lipocalin-2, IL-6, and TNF-α in sows and significantly negatively correlated with IL-10. Our findings support the hypothesis that intake of AM in the middle and late gestation period in sows reduces the occurrence of IUGR by alleviating maternal gut or systemic inflammatory responses and metabolic disorder.

In addition, increasing numbers of studies have found that gut microbiota are related to gut permeability and inflammation (40). Changes in the structural component of the diet, as the main energy source for gut microbiota, is an effective means of adjusting gut bacteria. Dietary fiber can not only regulate gut microbiota composition but also adjust microbial metabolites, including SCFAs, etc. Further, it can improve gut health and influence metabolism and animal behavior (41, 42). Zhao et al. reported that high dietary fiber could enrich 15 SCFAs produced by bacteria in the gut, stimulate SCFA production, improve the gut environment, reduce gut pH, increase butyrate concentration, competitively inhibit other “harmful bacterial,” and reduce the production of harmful metabolites (such as indoles and hydrogen sulfide) and thus build a healthier gut environment (43). Patients with irritable bowel syndrome have abnormal gut microbiota due to insufficient intake of SCFAs by gut epithelial cells, and the distribution of tight-junction proteins is directly affected, resulting in thinning of the gut microbiota, increased gut permeability, and decreased protective effects (44, 45). It has been suggested that dietary fiber-mediated changes in the gut microbiota and their metabolites may have important roles in maintaining a gut microecological balance and ensuring gut health. Our previous study (46) found that adding 5% AM to piglet diet could inhibit harmful bacteria, such as *Mycoplasma* and *Helicobacter* in piglet guts, and promote the proliferation of beneficial bacteria, such as *Paenibacillus*, *Lactococcus*, *Enterococcus*, and *Faecalibacterium*. Compared to the nonpregnant period, the physiological metabolic processes and immune systems of sows exhibit various changes in pregnancy to meet their physiological needs during this period. The gut microbiota changes significantly in different periods of pregnancy, which influences host metabolic processes, and the changes are related to the metabolic characteristics and immune system responses specific to the pregnancy period (47). Here, LEfSe analysis showed that the addition of AM to sow diets in mid and late pregnancy significantly increased the relative abundances of anti-inflammatory bacteria (*Prevotellaceae_NK3B31_group*, *norank_f_p_2534_18B5_gut_group*, *Lachnospiraceae_NK4A136_group*, and *g_Clostridium_sensu_stricto_1*) and decreased the relative abundance of proinflammatory bacteria (*Terrisporobacter*, *Desulfovibrio*, *Helicobacter*, *Eubacterium_fissicatena_group*, and *Erysipelotrichaceae_UCG_004*). Previous studies have found that colonization of *Clostridium_sensu_stricto_1* and other bacteria can promote the aggregation of CD4^+^ regulatory T cells in the colon of sterile mice and improve the level of transforming growth factor β, while early oral administration of *Clostridium* microbiota can increase resistance to colitis and systemic immunoglobulin. Colonization with *Clostridium* can also increase the colonization resistance of infant gut microbiota, hindering colonization of pathogenic bacteria (48); however, the colitis that may be caused by *Terrisporobacter* could promote gut microbiota malnutrition in animals (49). *Erysipelototrichaeae* and other bacteria can promote inflammation in patients with inflammatory bowel disease (50). *Helicobacter* is the main cause of chronic active gastritis and peptic ulcer (51). In addition, we found that the SCFA content in feces of sows fed with AM had an increasing trend, particularly butyrate at L4d, was significantly higher than the control. Previous studies have shown that SCFAs can promote the integrity of IL-10 epithelial cells and maintain gut homeostasis by inducing the GPR and NLRP3 inflammatory pathway (52). Further, butyrate can increase the anti-inflammatory ability of macrophages and dendritic cells by activating GPR109A, promoting regulatory T-cell differentiation, increasing the expression of the anti-inflammatory factor IL-10, and reducing the levels of the inflammatory factors IL-6 and IL-17 (53). Butyrate and propionate can reduce the likelihood of inflammatory bowel disease or colorectal cancer by inhibiting the differentiation of regulatory T cells induced by HDACs, maintaining the gut barrier, and controlling gut inflammation (54, 55). Our findings suggest that the addition of AM to the diet of sows in the mid and late gestation period can regulate gut microbiota and SCFA generation, thus improving sow gut health. Spearman correlation analysis further revealed that anti-inflammatory bacterial groups were positively correlated with anti-inflammatory factors, whereas proinflammatory bacterial groups were negatively correlated with proinflammatory factors. Moreover, the concentrations of acetic acid and butyric acid were positively correlated with anti-inflammatory bacteria of the *Lachnospiraceae_NK4A136_*group but negatively correlated with anti-inflammatory *Terrisporobacter* bacteria. Serum ROS levels were significantly positively correlated with proinflammatory factors and endotoxins but negatively correlated with anti-inflammatory factors, and there was a significant positive correlation between serum endotoxin and proinflammatory factors, as well as a significant positive correlation between fecal endotoxin concentration and zonulin. Furthermore, BA was significantly negatively correlated with proinflammatory factors, such as serum ROS and serum IL-6. Therefore, we conclude that the addition of AM in the middle and late gestation period of sows may improve disordered gut microbiota and decreased SCFAs generation, thus relieving the gut and systemic inflammatory response and promoting the healthy growth of sows.

**FIG 6:**
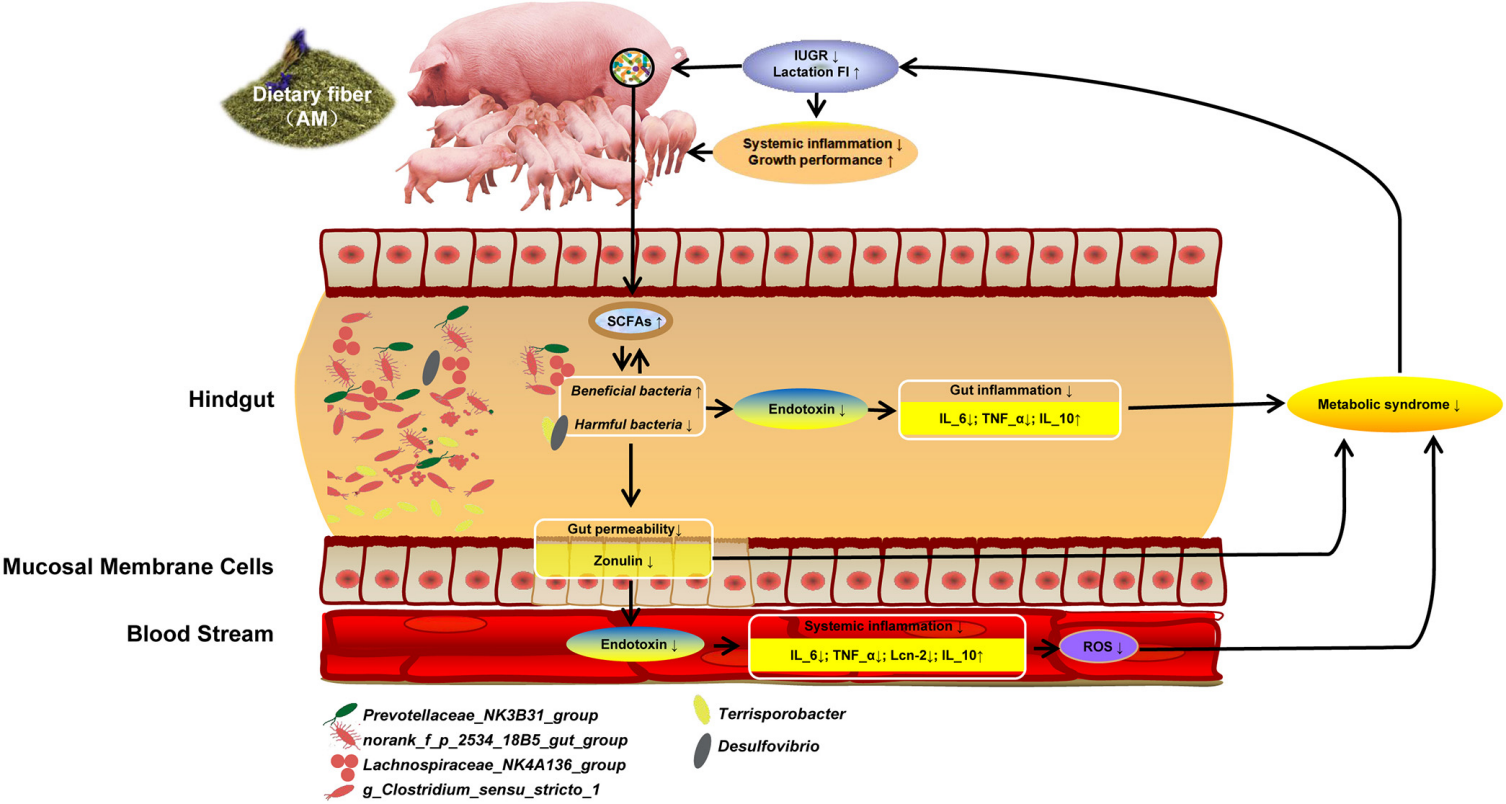
Systematic analysis of the effects of alfalfa meal diet on growth performance, inflammatory indexes, gut microbiota, and SCFAs of sows and piglets.

Using combined correlation analysis of growth performance, inflammatory indices, gut microbiota, and SCFAs in sows and piglets (Fig. 6), we propose that excessive ROS produced by overactive metabolic processes during late pregnancy and lactation will lead to increased endotoxin levels, disordered gut microbiota, decreased SCFA production, and secretion of proinflammatory factors, which will cause local inflammation of the gut, potential damage of the gut microbial barrier, increased gut permeability, increased blood endotoxin levels resulting in systemic inflammation, and ultimately, decreased sow and piglet performance. Supplementation of the diet with AM in mid and late pregnancy can reverse this process. Specifically, AM can increase the abundance of anti-inflammatory bacteria and reduce gut proinflammatory bacterial abundance by regulating the gut microbiota structure and SCFA production by AM fermentation, which decreases endotoxin and inflammatory factor secretion in the blood, resulting in reduced physiological inflammatory responses and improved sow performance, as well as reducing inflammatory responses in suckling piglets, and finally improving piglet gut health and growth performance. Nevertheless, further research is needed to elucidate the specific mechanisms underlying the interactions among gut microbiota, gut permeability, and the inflammatory and metabolic characteristics of sows and piglets.

In conclusion, we found that the addition of different fiber sources to the diet during mid and late gestation influenced the performance of sows and piglets. In particular, the addition of AM significantly improved sow and piglet performance and relieved gut and systemic inflammation. Furthermore, the supplementation with AM significantly increased the relative abundance of anti-inflammatory bacteria and decreased that of proinflammatory bacterial. We propose that the improvement in the performance of sows and piglets can be ascribed to the beneficial effects of AM on gut microbiota and the SCFA generation, resulting in decreased inflammatory responses and enhanced physical health in sows and piglets. These findings provide a theoretical basis and guide for the use of specific fiber sources in the diet of sows to improve gut health and production performance of sows and piglets. Our data also give insights for the study of the role of dietary fiber in the gastrointestinal function of human mothers and infants.

## MATERIALS AND METHODS

### Ethical approval.

All experimental procedures in this study were approved by the Institutional Animal Ethics Committee of Henan Agricultural University (approval HENAU-2018-015).

### Animals, diets, and housing.

Based on similar expected dates of confinement and backfat thickness, 48 sows (Large × Landrace) at 60 days of gestation were randomly allocated to the control (CK), alfalfa meal (AM), beet pulp (BP), and soybean skin (SH) groups. Each treatment included 12 replicated pens, each of which housed one sow. The preparation period was 7 days, and the test period was 75 days. All pregnant sows were supplied with feed formulated to meet National Research Council 2012 recommendations (56). The detailed ingredient composition and nutrient content of the investigated diets are presented in Table S1 in the supplemental material. On day 107 of pregnancy, sows were moved to individual farrowing pens with crates, slatted floors, and heat pads for the piglets. At parturition, the numbers of stillborn and live-born piglets in each litter were recorded. In the 12 h after farrowing, the litter size and individual piglet birth weights were measured. When possible, litter sizes were adjusted to 11 to 12 piglets, by adding or removing piglets within each dietary group without changing the mean litter birth weight. Lactating sows all consumed the same diet. Both sows and piglets had free access to water. The sow back fat thickness during the reproductive cycle and reproductive performance, as well as the growth performance of piglets, was recorded.

10.1128/mSystems.00591-20.2 TABLE S1 Ingredient and nutrient composition (%) of gestation diets. Download Table S1, DOCX file, 0.03 MB. Copyright © 2021 Liu et al. 2021 Liu et al. This content is distributed under the terms of the Creative Commons Attribution 4.0 International license.

### Sample collection.

At 100 days of gestation and at 4 and 18 days of lactation, four sows were selected from each treatment for collection of serum and fecal samples, and piglet blood samples were also collected at 4 and 18 days of lactation. Serum samples (5 ml) were collected in heparinized tubes from the vena jugularis of sows and piglets, with a minimal amount of stress. Plasma samples were then obtained by centrifuging the serum samples at 3,000 × *g* at 4°C for 10 min and stored at –80°C until analysis. Fresh fecal samples were collected individually from the pigs using sterile 20-ml centrifuge tubes and then stored at –80°C until analysis. According the performance indicators of sows, the optimal treatment was selected for the measurement of inflammatory factors, SCFA levels, and 16S rRNA gene sequencing.

### Measurement of inflammatory factors.

IL-6, IL-10, TNF-α, endotoxin, zonulin, lipocalin-2, and ROS were measured in sow serum samples, and IL-6, IL-10, TNF-α, and endotoxin in sow stool samples. IL-6, IL-10, TNF-α, endotoxin, and ROS were also measured in piglet serum samples. Inflammatory factors were evaluated using enzyme-linked immunosorbent assay technology (Nanjing Jiancheng Bioengineering Institute, Nanjing, China). All procedures were performed in duplicate.

### Determination of SCFAs levels.

Gas chromatography (GC) performed as described by Liu et al. (46) was used to determine the SCFA levels of in stool samples. The samples were analyzed on an HP-88 column (100-m length, 0.25-mm diameter, and 0.2-μm film thickness from the producer) and separated using a TRACE 1310 GC with a flame ionization detector. The temperature program was as follows: 70°C for 1 min, followed by an increase to 180°C held at 25°C for 1 min, an increase to 200°C held at 10°C for 1 min, an increase to 220°C held at 2°C for 10 min, and finally an increase to 240°C held at 20°C for 6 min. The sample was run with a split ratio of 20:1 and a column flow rate of 1.3 ml/min. Hydrogen is used as a carrier gas. The injector temperature is 270°C, and the detector temperature is 290°C.

### DNA extraction and 16S rRNA gene sequencing.

Microbial DNA was extracted from feces samples by using an E.Z.N.A. soil DNA kit (Omega Bio-Tek, Norcross, GA) according to the manufacturer’s protocols. The final DNA concentration and purity were determined by using a NanoDrop 2000 UV-vis spectrophotometer (Thermo Scientific, Wilmington, DE), and the DNA quality was checked by 1% agarose gel electrophoresis. The V3-V4 hypervariable regions of the bacterial 16S rRNA gene were amplified using the primers 338F (5′-ACTCCTACGGGAGGCAGCAG-3′) and 806R (5′-GGACTACHVGGGTWTCTAAT-3′) by PCR (GeneAmp 9700; ABI) (57), with the following program: 3 min of denaturation at 95°C; 27 cycles of 30 s at 95°C, 30 s of annealing at 55°C, and 45 s of elongation at 72°C; and a final extension at 72°C for 10 min. PCRs were performed in triplicate, with each 20-μl reaction mixture containing 4 μl of 5× FastPfu buffer, 2 μl of 2.5 mM deoxynucleoside triphosphates, 0.8 μl of each primer (5 μM), 0.4 μl of FastPfu polymerase, and 10 ng of template DNA. The resulting PCR products were extracted from 2% agarose gels, further purified using the AxyPrep DNA gel extraction kit (Axygen Biosciences, Union City, CA), and quantified using a QuantiFluor-ST instrument (Promega) according to the manufacturers’ protocols. Purified amplicons were pooled in equimolar amounts and subjected to paired-end sequencing (2 × 300 bp) on an Illumina MiSeq platform (Illumina, San Diego, CA), according to standard protocols, by Majorbio Bio-Pharm Technology Co., Ltd. (Shanghai, China).

### Bioinformatics analysis of sequencing data.

Raw fastq files were demultiplexed, quality filtered using Trimmomatic, and merged using FLASH, according to the following criteria: (i) reads were truncated at any site receiving an average quality score of <20 over a 50-bp sliding window; (ii) primers were exactly matched, allowing 2-nucleotide mismatching, and reads containing ambiguous bases removed; and (iii) sequences whose overlap was longer than 10 bp were merged, according to their overlap sequence. Operational taxonomic units (OTUs) were clustered with a 97% similarity cutoff using UPARSE (v7.1 [http://drive5.com/uparse/]), and chimeric sequences identified and removed using UCHIME. The taxonomy of each 16S rRNA gene sequence was analyzed using the RDP Classifier algorithm (http://rdp.cme.msu.edu/) against the Silva (SSU128) 16S rRNA database, with a 70% confidence threshold. Sample biodiversity was calculated using the ACE, Chao1, and Shannon indices by applying a Wilcoxon rank sum test. Beta-diversity measures dependent on Bray-Curtis and unweighted-UniFrac distance values were calculated using mothur. LEfSe analysis was conducted to identify bacterial taxa differentially represented between different groups at the phylum to genus taxonomy level (biomarkers) by one-against-all (less strict). To determine the effect of microbiota interacting with Apparent performance, redundancy analysis (RDA) was performed at the genus level using the R language vegan packet on Spearman correlation analysis (RDA 2014).

### Statistical analysis.

Statistical analyses were performed using SPSS 20.0 software (IBM, New York, NY). Data were evaluated by one-way ANOVA, and the differences between means assessed using Duncan’s test. A *P* value of <0.05 was considered statistically significant. The data were evaluated by Spearman correlation analysis of the Euclidean distance.

### Data availability.

Raw reads were deposited into the NCBI Sequence Read Archive database under accession number SRP268238.
