# Supplementary figures and images for: Consumption of Dietary Fiber from Different Sources during Pregnancy Alters Sow Gut Microbiota and Improves Performance and Reduces Inflammation in Sows and Piglets
Source: mSystems. 2021 Jan 26;6(1):e00591-20. doi: 10.1128/mSystems.00591-20 (PMC7842364; doi:10.1128/mSystems.00591-20)

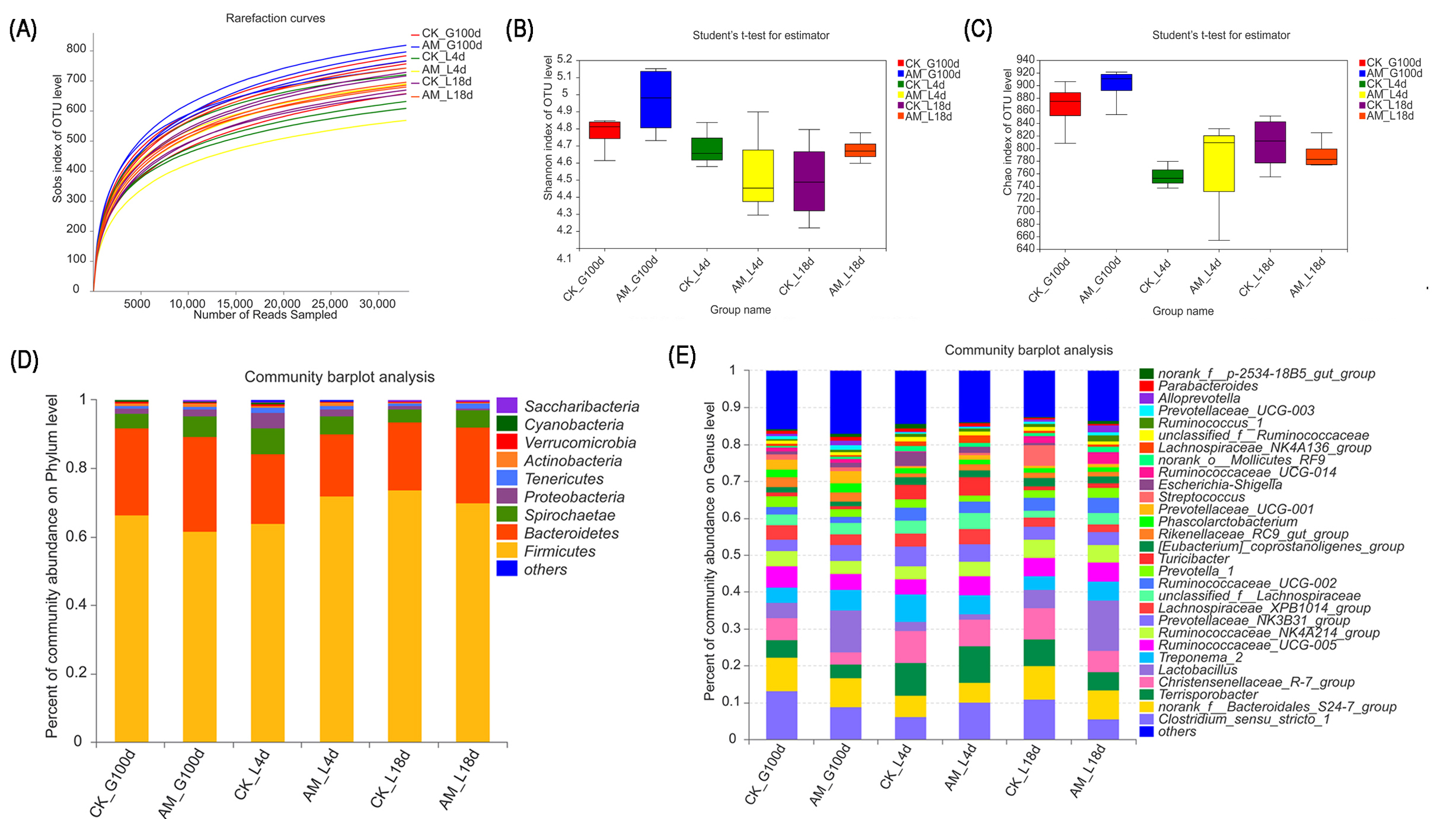

Supplement: FIG S1 [file mSystems.00591-20-sf001.jpg]
