# Supplementary material for: Consumption of Dietary Fiber from Different Sources during Pregnancy Alters Sow Gut Microbiota and Improves Performance and Reduces Inflammation in Sows and Piglets
Source: mSystems. 2021 Jan 26;6(1):e00591-20. doi: 10.1128/mSystems.00591-20 (PMC7842364; doi:10.1128/mSystems.00591-20)
Supplement: TABLE S1 [file mSystems.00591-20-st001.docx]

# Supplementary Table

**Table S1 Ingredient and nutrient composition of gestation diets %**

| Items | CK^a^ | AM^a^ | BP^a^ | SH^a^ |
| --- | --- | --- | --- | --- |
| Ingredient (%) |  |  |  |  |
| Corn | 591.6 | 321.8 | 448.5 | 317 |
| Soybean meal | 96.4 | 46.5 | 98.5 | 47.5 |
| Wheat bran | 240.3 | 242.3 | 297.3 | 133.5 |
| Wheat middling | 38 | 216.5 | 0 | 388.3 |
| Alfalfa meal |  | 100 |  |  |
| Beet pulp |  |  | 100 |  |
| Soybean skin |  |  |  | 80 |
| Soybean oil |  | 39.2 | 22 |  |
| Limestone | 12.88 | 12.88 | 12.88 | 12.88 |
| CaHPO4 | 6.82 | 6.82 | 6.82 | 6.82 |
| Nacl | 4 | 4 | 4 | 4 |
| Premix | 10 | 10 | 10 | 10 |
| Total | 1000 | 1000 | 1000 | 1000 |
| Nutrient composition |  |  |  |  |
| DE^b^ (Mcal/kg) | 3.05 | 3.04 | 3.05 | 3.05 |
| CP^b^ (%) | 14.00 | 14.01 | 14.01 | 14.01 |
| CF^b^ (%) | 3.48 | 5.16 | 5.15 | 5.16 |
| NDF^b^ (%) | 16.01 | 20.12 | 20.13 | 20.11 |
| ADF^b^ (%) | 5.87 | 7.95 | 8.01 | 8.00 |
| Ca^b^ (%) | 0.78 | 0.87 | 0.89 | 0.77 |
| P^b^ (%) | 0.57 | 0.61 | 0.58 | 0.55 |
| Lys^b^ (%) | 0.68 | 0.62 | 0.71 | 0.63 |
| Met^b^＋Cys^b^ (%) | 0.47 | 0.47 | 0.47 | 0.50 |
| Thr^b^ (%) | 0.49 | 0.49 | 0.51 | 0.48 |

^a^ Control group (CK), Alfalfa meal group (AM), Beet pulp group (BP), Soybean skin group (SH)

^b^ DE: Digestion energy, CP: Crude protein, CF: Crude fiber, NDF: Neutral detergent fibre, ADF: Acid Detergent Fiber, Ca: Calcium, P: Phosphorus, Lys: lysine, Met: Methionine, Cys:Cysteine, Thr: Threoni
